# Chronic care management of globesity: promoting healthier lifestyles in traditional and mHealth based settings

**DOI:** 10.3389/fpsyg.2015.01557

**Published:** 2015-10-15

**Authors:** Gianluca Castelnuovo, Giada Pietrabissa, Gian Mauro Manzoni, Stefania Corti, Martina Ceccarini, Maria Borrello, Emanuele M. Giusti, Margherita Novelli, Roberto Cattivelli, Nicole A. Middleton, Susan G. Simpson, Enrico Molinari

**Affiliations:** ^1^Psychology Research Laboratory, Istituto Auxologico Italiano, Istituti di Ricovero e Cura a Carattere Scientifico, Ospedale San Giuseppe, Verbania, Italy; ^2^Department of Psychology, Catholic University of Milan, Milan, Italy; ^3^Faculty of Psychology, eCampus University, Milan, Italy; ^4^Department of Psychology, University of Bergamo, Bergamo, Italy; ^5^School of Psychology, Social Work and Social Policy, University of South Australia, Adelaide, SA, Australia

**Keywords:** obesity, type 2 diabetes, rehabilitation, mHealth, clinical psychology

## Abstract

Obesity and being overweight could be real chronic conditions above all if there are other complications such as type 2 diabetes, cardiovascular diseases, hypertension, dyslipidemia, hypercholesterolemia, cancer, and various psychosocial and psychopathological disorders. Due to the multifactorial etiology of obesity, evidence-based interventions to improve weight loss, maintain a healthy weight, and reduce related comorbidities combine different treatment approaches: dietetic, nutritional, physical, behavioral, psychological, and, in some situations, pharmacological and surgical. There are significant limitations in this multidisciplinary chronic care management of obesity, most notably those regarding costs and long-term adherence and efficacy. Programs including eHealth platforms and new technologies could overcome limitations connected to the traditional in-patient chronic care management of obesity, thus providing promising opportunities in enhancing weight reduction and reducing complications in terms of long-term efficacy and effectiveness across clinical, organizational, and economic perspectives.

## A Multidisciplinary Chronic Care Management of Globesity

“Globesity” could be defined as a new global epidemic of obesity and being overweight with many associated complications and chronic conditions. Such conditions include not only type 2 diabetes, but also cardiovascular diseases, hypertension, dyslipidemia, hypercholesterolemia, cancer, and various psychosocial and psychopathological disorders (Wadden et al., 2002; Byrne et al., 2004; Flegal et al., 2005; Whitlock et al., 2009; Castelnuovo et al., 2014).

The etiology of obesity is universally recognized as multifactorial with a complex interaction between genetic, behavioral and environmental factors (Marcus and Wildes, 2009). Genetics plays an important role, but behavioral factors, such as dysfunctional eating habits and low levels physical activity, are among the main modifiable and proximal causes strictly connected to obesity and obesity-related complications (Dombrowski et al., 2012).

Due to the multifactorial etiology of obesity, evidence-based interventions to improve weight-loss, maintain a healthy weight, and reduce related comorbidities combine different treatment approaches: dietetic, nutritional, physical, behavioral, psychological, and, in some situations, pharmacological and surgical.

Clinical interventions, which typically focus on weight loss, reduction of obesity-related comorbidities, and change in dysfunctional behaviors, should be implemented in a multidisciplinary context with a clinical team composed of endocrinologists, nutritionists, dieticians, physiotherapists, psychiatrists, psychologists, and sometimes surgeons.

Also social and environmental barriers have to be considered in order to promote more effective strategies in weight loss programs (Rosas et al., 2015). For example policy interventions can potentially promote healthy behaviors (functional eating, physical activity) at the population level and not only in clinical settings (Barnidge et al., 2013). The socioeconomic situation of the patient has to be considered, in fact in developed countries the spread of obesity is more relevant than in lower socio-economic subgroups. Some specific reasons have been underlined by Mauro et al. (2008): “This has been attributed to the greater density of fast-food restaurants in low-income neighbourhoods…, higher cost of healthy diets…, safety concerns that prevent walking and other outdoor activities… and greater social acceptance of excess body weight…. Affordability of membership in commercial weight-loss programs, gyms, obesity medications or surgery can likewise prove to be important obstacles. All of these factors can pose important barriers to weight management and interventions must specifically acknowledge and address these limitations” (p. 173). About child obesity, parents underline different obstacles to promote obesity prevention guidelines, such as child preferences, resistance to change habits, lack of knowledge and lack of a permanent monitoring attitude toward child behaviors, etc. (Sonneville et al., 2009).

Psychosocial and psychopathological determinants are key elements to consider in the successful long-term treatment of obesity due to the significant correlations between being overweight and self-esteem, quality of life, stress, life events, family and systemic scenarios, eating disorders, mood disorders, anxiety disorders, and personality disorders (Hudson et al., 2007; Pickering et al., 2007; Petry et al., 2008; Scott et al., 2008; Davin and Taylor, 2009; Manzoni et al., 2010).

For the in-patient management of obesity with or without type 2 diabetes, many psychological treatments are available, such as psychoeducational, cognitive-behavioral, interpersonal, systemic-strategic, and psychodynamic (Shaw et al., 2005).

Psychological therapies, typically focusing on dysfunctional behaviors, cognitive processes, unrealistic weight goals, and body image perceptions, could better help patients in achieving weight loss outcomes, both inside hospitals and clinical centers and during out-patient follow-up sessions (Wing, 2002; Swencionis and Rendell, 2012).

Some psychological skills should be implemented for functional chronic care management of obesity and its complications. Such skills include determining the client’s ability to self-monitor (e.g., using diaries), assistance with stimulus control through restricting quantities of food, and behavioral modification strategies (e.g., chewing slowly, taking time to enjoy food, and increasing awareness of the pleasure associated with taste and food; Wing, 2002; Foster et al., 2005; Swencionis and Rendell, 2012). Specific psychological actions are also required in order to maintain goals that have initially been achieved, manage possible relapses, and learn strategies to cope with critical situations (Manzoni et al., 2010, 2011b; Dombrowski et al., 2011, 2012; Capodaglio et al., 2013).

## High Costs and Long-term Problems of Adherence in Traditional in-patient Chronic Care Management of Globesity

Significant limitations in the multidisciplinary chronic care management of globesity concern costs and long-term adherence and efficacy. The obesity epidemic has been historically considered as a disease of high-income countries, but now is clearly characterizing also low- and middle-income countries with an increasing economic burden (Mitchell and Shaw, 2015). Although this scenario is critical, lifestyle interventions show promising results in terms of clinical evidence and cost-effectiveness (Nugent, 2008; Boyers et al., 2015; Jerome et al., 2015; Whitfield et al., 2015).

Another limitation is the difficulty associated with maintaining long-term compliance and adherence in order to ensure clinical efficacy. “In fact, most overweight and obese individuals regain about one third of the weight lost with treatment within one year and they will typically come back to baseline in three to five years” (Pietrabissa et al., 2012, p. 317). Specifically in relation to the presence of diabetes complication, “Lifestyle intervention has been effective in several countries, but its success depends on uptake of intervention programmes and on compliance… An urgent priority is to identify ways to effectively engage people at risk of diabetes. Long-term sustainability is also a concern” (Zimmet et al., 2014, pp. 61–62).

Assessment of patients’ motivation, compliance, and engagement is a key factor of treatment for obesity and its comorbidities (Waller et al., 2011; Barello et al., 2012). For example, the transtheoretical model of change (Prochaska and Diclemente, 1984; Astroth et al., 2002), which describes five motivational stages through which patients necessarily evolve while trying to change their dysfunctional behaviors, could be useful in explaining and predicting how and when individuals change their own unhealthy behaviors (Sarkin et al., 2001). Motivational Interviewing, defined as a “client-centred, directive method for enhancing intrinsic motivation to change by exploring and resolving ambivalence” (Wilson and Schlam, 2004), and Motivational Enhancement Therapy are other potential steps forward in comparison with the transtheoretical model (Miller and Johnson, 2001).

## New Technologies for Chronic Care Management of Globesity: The mHealth Perspective

mHealth (also m-health, mHealth, or mobile health) could be defined as the practice of medicine and public health supported by mobile communication devices, such as mobile phones, tablet computers, and personal digital assistants (PDAs), for health services and information (Eysenbach, 2001, 2011; Riper et al., 2010; Cipresso et al., 2012; Whittaker, 2012; Fiordelli et al., 2013; Castelnuovo et al., 2014, 2015).

Programs with eHealth platforms and new technologies could overcome limitations connected to the traditional in-patient chronic care management of obesity with type 2 diabetes by providing promising opportunities for enhancing weight reduction and reducing complications in terms of long-term efficacy and effectiveness across clinical, organizational, and economic perspectives (Khaylis et al., 2010; Manzoni et al., 2011a; Rao et al., 2011). These technology-based strategies provided in out-patient settings are based on a collaborative approach derived from central planning and grounded in chronic care logic (Rao et al., 2011).

Particularly, considering the specific situation of obesity with type 2 diabetes, a detailed description of all possible mHealth applications is reported in Chomutare et al. (2011), where authors found more than 260 different diabetes applications (for Apple iPhone, Google Android, BlackBerry, and Nokia Symbian). All apps focused on the following features and measurements: self-monitoring, blood glucose, weight, physical activity, diet, insulin and medication, blood pressure, education, disease-related alerts and reminders, integration of social media functions, disease-related data export and communication, synchronization with personal health record (PHR) systems, and patient portals (Chomutare et al., 2011). Even if these apps are promising, new technologies have not yet demonstrated enough evidence in comparison with traditional approaches: for example computer-based self-management interventions for type 2 diabetes have only a limited beneficial effect on blood glucose control (a positive result is that this effect was more significant considering the mobile phone subgroup; Pal et al., 2013). Moreover these technologies have not yet produced significant impacts in other medical and psychological (cognitive, behavioral, emotional) variables (Pal et al., 2013).

Many clinical applications have been published about the utility of mobile phone devices in promoting healthy habits, weight loss attitudes, and reduction of comorbidities (Castelnuovo et al., 2010; Chomutare et al., 2011; Manzoni et al., 2011a; Rao et al., 2011; Simpson and Slowey, 2011; Burke et al., 2012; Cafazzo et al., 2012; Park and Kim, 2012; Pellegrini et al., 2012; Schiel et al., 2012; Bacigalupo et al., 2013; Fiordelli et al., 2013; Hebden et al., 2013; Martinez-Perez et al., 2013; Rodrigues et al., 2013; Schoffman et al., 2013; Sharifi et al., 2013; Shaw et al., 2013; Turner-McGrievy et al., 2013). One interesting project was the POWeR (“Positive Online Weight Reduction”) Web-based weight management intervention that underlined the importance of supplementing new technologies based obesity treatment protocols with brief human support (Dennison et al., 2014). The mHealth approach has shown positive evidence not only in adult obesity (Tufano and Karras, 2005; Burke et al., 2012), but also in pediatric obesity (Jensen et al., 2012; Turner-McGrievy et al., 2013). Applications have also been shown to increase participation, compliance, and engagement (Graffigna et al., 2013a,b).

Khaylis underlined five psychological components (Self-monitoring, Counselor feedback and communication, Social support, Structured program, Individually-tailored program) necessary for functional mHealth-based chronic case management in order to facilitate weight loss and a reduction of comorbidities such as type 2 diabetes (Khaylis et al., 2010).

## Barriers to the Expansion of mHealth Chronic Care Management of Globesity

Technical problems, skepticism, and reticence from patients, caregivers, nurses, and physicians could limit the growth of mHealth solutions (Gaggioli et al., 2005; Rees and Stone, 2005). Unfortunately, data collected about real costs of telemedicine are conflicting. Even so, mHealth could certainly reduce travel time, hospital admissions, and indirect costs for service users and their families and social networks.

Mohammadzadeh and Safdari (2014) summarized the main barriers and challenges in the development of mHealth scenarios in chronic care management of different pathologies:

• Organizational and technological barriers
• User attitudes
• Technology acceptance
• Threats to confidentiality and privacy
• Legal, ethical, and administrative barriers
• Costs of system implementation
• Costs of system maintenance
• Lack of sufficient investment
• Poor design and implementation
• Lack of system interoperability with electronic health records and other IT tools
• Decrease in face-to-face communication between doctor and patient
• Poor functioning of system that leads to medical errors and negative effects on care outcomes, patients and personnel
• Mistakes in documentation; misrepresentation
• Data manipulation and violation of patients’ legal rights
• Reliability, sustainability of connections, sudden interruptions of telecommunication networks
• Scalability in terms of data rate and power and energy consumption
• Antenna design, quality of service (QoS), energy efficiency
• Weight of wearable devices
• Training user to use wearable system
• Wearable system market penetration

## Future Directions in Clinical Psychology and Medicine for Chronic Care Management of Globesity

Turner-McGrievy noted that potential benefits of mobile monitoring methods for behavioral weight loss protocols appear clear: “future studies should examine ways to predict which self-monitoring method works best for an individual to increase adherence” (Turner-McGrievy et al., 2013, p. 513). There is a critical need for scientific research to evaluate the specific outcomes of collaborative approaches for weight management that utilize the Internet and mobile-based tools.

The mHealth approach could help clinicians by motivating patients in remote settings to develop healthier lifestyles (Pietrabissa et al., 2012), to accept more intrusive medical treatments, and to cope with chronic conditions by reducing complications (such as type 2 diabetes, hypertension, and cardiovascular disease; Nguyen and Lau, 2012).

Further studies are required to evaluate the feasibility of the chronic care model as a perspective in obesity interventions (Rao et al., 2011). It is imperative that clinical psychology and medicine develop new diagnostic, monitoring and treatment protocols usable in both traditional and innovative technology-based settings, incorporating recent scientific and clinical evidence (Pietrabissa et al., 2012) and respectful communication (Kahn, 2008; Macagno and Walton, 2010; Bigi, 2011; Castelnuovo, 2013) even in an mHealth scenario.

One promising future direction could be treating obesity, considered as a chronic multifactorial disease, using a stepped-care approach (Kushner, 2014; Castelnuovo et al., 2015):

• The lower level of treatment could be simply a *mHealth or traditional based lifestyle psychoeducational and nutritional approach* to weight management. This step could be also considered as a prevention phase at the population level.
• The following step, useful in moderate conditions, could be represented by the inclusion in *health professionals-driven multidisciplinary protocols* tailored for each patient. The mHealth approach could be useful for providing many parts of the clinical program reducing costs and limiting hospitalizations.
• Another step of care, useful in severe conditions, could be the *inpatient approach with the inclusion of drug therapies and other multidisciplinary tretaments* if necessary. The mHealth contribution could be useful for monitoring and motivating patients after the inpatient phase and for reducing costs above all in the follow-up steps.
• Another step, in more severe conditions, needs the solution of *bariatric surgery* and with this option the mHealth approach could be useful for monitoring eating attitudes, motivating patients in changing dysfunctional behaviors and for reducing costs above all in the follow-up steps.

In the chronic care management of globesity mHealth solutions cannot substitute traditional approaches, but they can supplement some steps in obesity prevention and weight loss management, above all in the follow-up phase where the mobile technology can ensure a continuity of care saving costs and avoiding long term lack of connections between patients-citizens and the health care team.

### Conflict of Interest Statement

The authors declare that the research was conducted in the absence of any commercial or financial relationships that could be construed as a potential conflict of interest.
